# Distinct spatial and temporal roles for Th1, Th2, and Th17 cells in asthma

**DOI:** 10.3389/fimmu.2022.974066

**Published:** 2022-08-12

**Authors:** Weihang Luo, Jindong Hu, Weifang Xu, Jingcheng Dong

**Affiliations:** ^1^ Department of Integrative Medicine, Huashan Hospital, Fudan University, Shanghai, China; ^2^ Institutes of Integrative Medicine, Fudan University, Shanghai, China; ^3^ Shenzhen Hospital of Guangzhou University of Chinese Medicine (Futian), Shenzhen, China

**Keywords:** asthma, T helper cells, Th1, Th2, Th17, cytokines, pathogenetic T cells, CD4^+^ memory T cell

## Abstract

Immune response in the asthmatic respiratory tract is mainly driven by CD4^+^ T helper (Th) cells, represented by Th1, Th2, and Th17 cells, especially Th2 cells. Asthma is a heterogeneous and progressive disease, reflected by distinct phenotypes orchestrated by τh2 or non-Th2 (Th1 and Th17) immune responses at different stages of the disease course. Heterogeneous cytokine expression within the same Th effector state in response to changing conditions *in vivo* and interlineage relationship among CD4^+^ T cells shape the complex immune networks of the inflammatory airway, making it difficult to find one panacea for all asthmatics. Here, we review the role of three T helper subsets in the pathogenesis of asthma from different stages, highlighting timing is everything in the immune system. We also discuss the dynamic topography of Th subsets and pathogenetic memory Th cells in asthma.

## Introduction

Asthma is a heterogeneous disease characterized by chronic airway inflammation and airway hyperresponsiveness (AHR), resulting in repeated periods of symptoms that include wheezing, shortness of breath, and cough. Asthma often starts in early childhood and then develops progressively due to exacerbations caused by a respiratory viral infection or inappropriate treatment. The pathogenesis of asthma is closely related to mucus overproduction, activation of inflammatory cells, airway remodeling, and airway narrowing, which is the consequence of complex interaction between epithelial cells and immune cells. Among these immune cells, Th2 cells have long been considered the primary culprit in the development of asthma. However, the role of Th1 and Th17 cells as a friend or a foe in asthma has long been puzzling. The eosinophilic inflammation in the airway mediated by Th2 cells could be attenuated by the administration of pro-Th1 or Th17-associated cytokines, including IL-12 and IL-17 (1, 2). Nevertheless, the biological anti-Th2 signaling is not the panacea for all Th2-high asthmatics. It has also been found that Th1 and Th17 signatures occur in some individuals with severe asthma (3).

The canonical model of CD4^+^ T cell differentiation derived from a simple well-defined culture conditions has occupied our minds for more than the last 30 years laying a solid foundation for the classical Th1/Th2 balance model in which asthma is considered to be the result of Th2-skewed immune response. However, this model has been an oversimplification, and in fact, immune response *in vivo* is much more nuanced than anticipated, involving a greatly heterogeneous pool of Th cell subpopulations with different cytokine expression patterns that have the potential to fine-tune their response according to the dynamic inflammatory milieu in different tissues and times. The timing and functional nuances of cytokine expression profile within Th cell subpopulations can differentiate between a friend that is typically immunosuppressive phenotype and a foe that is principally the terminal differentiation of phenotype with atypical expression of cytokines. In this review, we will discuss the kinetics of the Th1, Th2, and Th17 immune responses incorporating temporal and spatial cues in the context of asthma.

## The differentiation of CD4^+^ T cells

Th1 and Th2 cells were recognized in the late 1980s, when Mosmann and Coffman noted that there existed two distinct cytokine expression profiles among CD4^+^ T cells, eliciting two mutually exclusive immune responses (4). IL-12, the critical cytokines for Th1 cell differentiation, promotes activation of signal transducer and activator of transcription 4(STAT4)that subsequently induces the transcription factor T-bet to activate Th1 cells (5). Similarly, IL-4 promotes activation of STAT6 that rapidly induces transcription factor GATA3 to activate Th2 cells (6) In 2005, Seminal work by two groups identified a true distinct lineage of CD4^+^ T cells named Th17 cell, whose developmental programs are distinguished from Th1 and Th2 cells (7, 8). τh17 cells are characterized by the production of IL-17 and IL-22 as signature cytokines and expression of Retinoic acid (RA)-related orphan receptor γ (RoRγt) as the master transcription factor (9).

### The common differentiation module for CD4+ T cells

**Figure 1:**
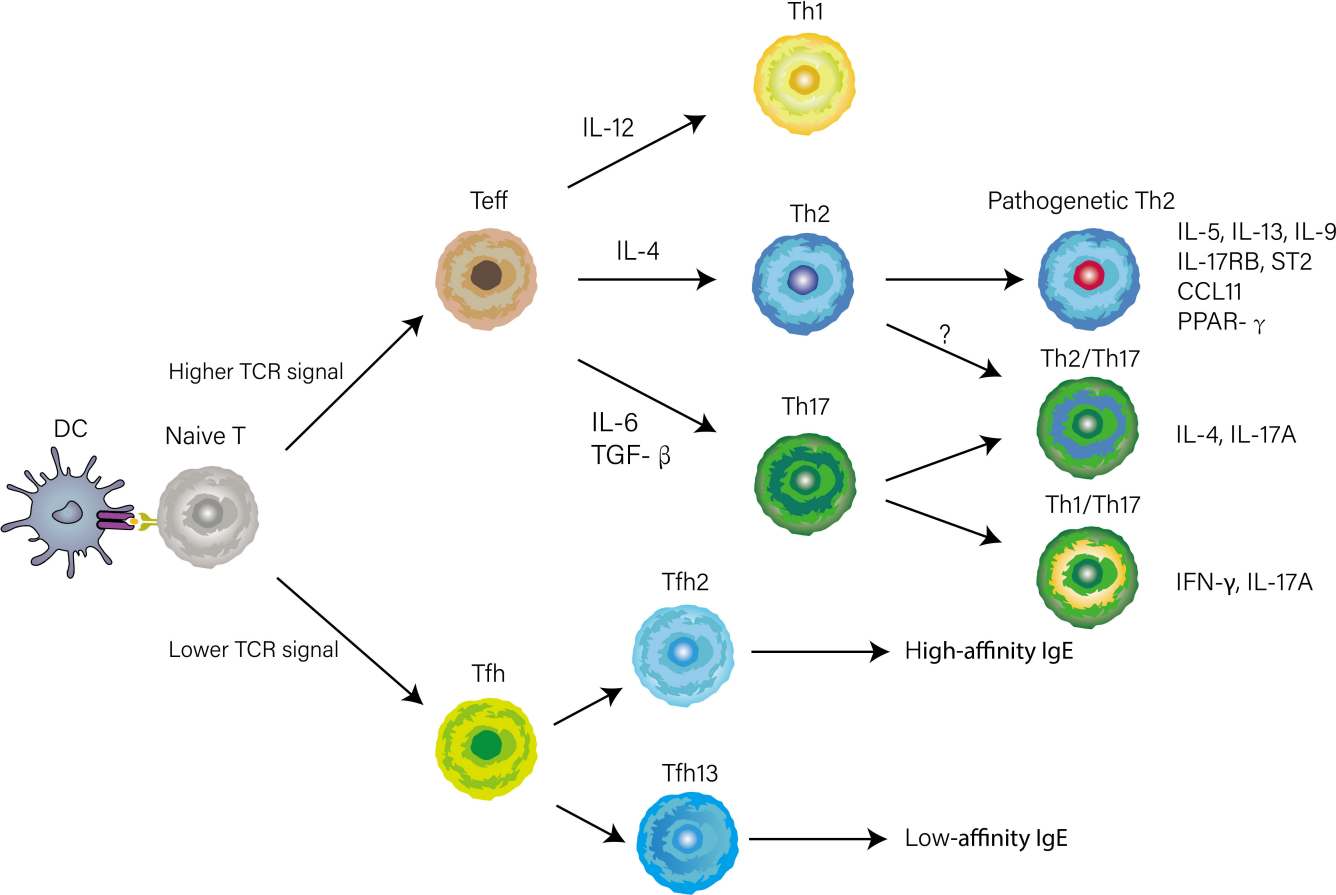
CD4^+^ T cells differentiation and heterogeneity in the context of asthma. The early differentiation is primed by antigen presentation of DCs in lymphoid tissue. Whether naïve T cell differentiates into Tfh or Teff cell is determined by TCR signal strength. When stimulated by a high TCR signal, naïve T cells differentiate into Teff cells. Concurrently, Innate cytokines direct functionally distinct effector programs (e.g., Th1, Th2, and Th17) by inducing lineage-defining transcriptional networks. Several phenotypically and functionally CD4^+^ T cells have recently been identified based on the expression of cytokines, transcription factors, or surface markers.

With the appearance of *in vivo* experimental systems, our understanding of CD4^+^ T cell differentiation, largely based on reductionist cell culture experiments, has altered. Herein we break with the traditional paradigm and describe complex fate-determining transcriptional networks directing Th1, Th2, and Th17 cell differentiation in the lymph nodes and non-lymphoid tissues according to the experimental system *in vivo*. Early works attempted to control the polarization by changing the intensity of T cell antigen receptor (TCR) stimulation and the combination of cytokines in the medium, to elucidate the key cues that regulate CD4^+^ T cell differentiation (10). However, the temporal and spatial factors were not taken into account in these studies, thus provoking a question of whether or not these cues direct CD4^+^ T cell differentiation in the complex microenvironment of lymph nodes and lungs. Recent work has identified newly Th2 subsets displaying pathogenetic phenotypes which were not induced *in vitro* culture conditions, such as peTh2 and Th2A cells, further implying there were apparent limitations in early studies due to ignoring the complex microenvironment of the individual that changes over time and space (11, 12). Ruterbusch and colleague proposed the modules of CD4^+^ T cells differentiation that merges at least two dimensionalities of heterogeneity: subpopulations heterogeneity, which comprises Th1, Th2, and Th17 cells directed by cytokines produced by innate immunity; activation state, which consists of high-cytokine-producing effector Th cell or B cell-helping T follicular helper (Tfh) cell mainly depending on TCR signal stimulation (**Figure 1**) (13). Once CD4+ T cells are primed on Dendritic cells (DCs) and acquire high TCR signaling, they can differentiate into Teff cells and home to the lung, where they integrate tissue-derived cues to fine-tune their function. Meantime activated Tfh cells through low TCR stimulation migrate to the T-B border in the lymphoid tissue. Distinguished from previously described Teff cell subsets, Tfh cells are newly identified subsets of CD4^+^ helper cells, and Bcl-6 is a fate-determining signature transcription factor essential for their differentiation (14–16). A recent report found that IL-4-producing Tfh (Tfh2) cells, a well-known contributor to induction of IgE, are precursors of effector Th2 cells (17). Intriguingly, a subsequent study describes a subset of IL-4 and IL-13-producing Tfh cells named Tfh13 cells that are indispensable for the induction of high-affinity IgE (**Figure 1**) (18). The bifurcated but parallel interaction between Tfh13 and effector Th2 cells orchestrates the humoral and cellular immunity in asthma (19). In the context of bacterial infections, such as Streptococcus pyogenes or Listeria monocyte, CD4^+^ T cells with high expression of CD25 that are deviated from Tfh lineage commitment would then become Th1 or Th17 cells depending on innate cell-derived cytokines (20, 21).

### The differentiation of Th1 and Th2 cells

**Figure 2:**
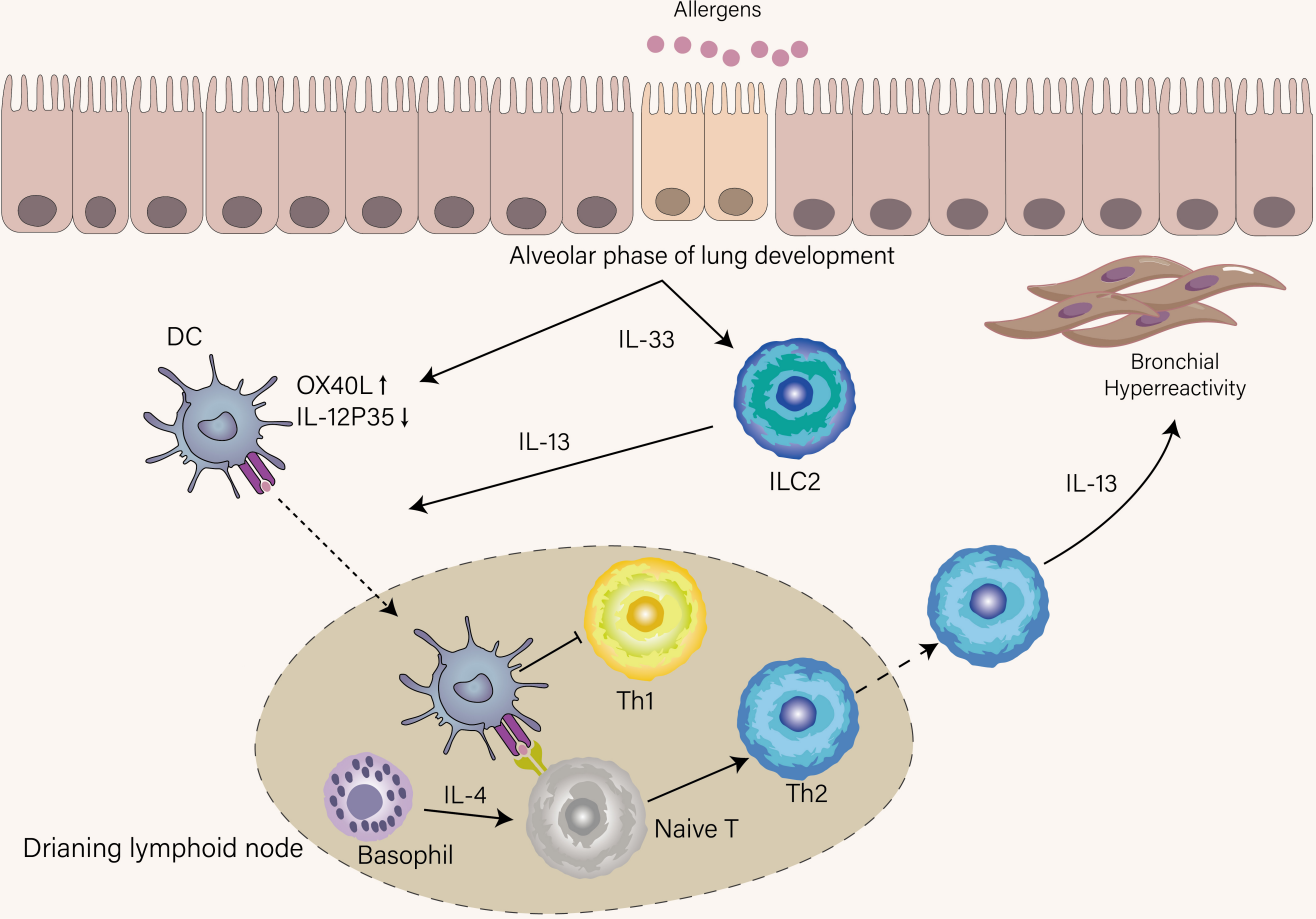
Mechanisms that condition inception of early-life asthma. IL-33 was spontaneously produced by epithelial cells until the age of three years, a period termed the alveolar phase of lung development. Under the influence of IL-33, DCs upregulate OX-40L and suppress IL-12p35, thus favoring Th2 cell skewing, and ILC2s were accumulated and activated in the lung. ILC2s provide IL-13 to boost DCs that recognize allergens to migrate into lymphoid tissue. DCs in lymphoid tissue promote Th2 differentiation with the help of IL-4 derived from basophils and simultaneously inhibit Th1 differentiation. IL-13 derived from Th2 induces AHR, which is a hallmark of recurrent wheeze in children who develop asthma.

Compared with the Th1 response, where intracellular pathogens directly activate IL-12-producing DCs to induce Th1 cell differentiation, the initiation of the Th2 response is indirect by DCs and poorly understood. Upon airway epithelia encountering inhaled aero-allergen, these stimuli induce the release of IL-33 from epithelia, which activates DCs, basophils, and group 2 innate lymphoid cells (ILC2s) (22). Stimulated ILC2s secrete IL-13 to promote activated DCs migration into the draining lymph node (23). If DCs can give strong TCR stimulation to naïve T cells in the presence of IL-4 that is derived from basophils recruited into lymph nodes (24), naïve T cells will differentiate into effector Th2 cells that express high levels of GATA-3, STAT6, and Blimp-1 and home to the lung (**Figure 2**). Otherwise, in the same cytokine milieu, a naïve clone migrates into the T-B border as a Tfh13 cell to produce high-affinity IgE, leading to immediate degranulation of mast cells (18).

Since the chronic inflammation of asthma is mainly located in the airway, those effector Th2 cells that migrate out of the draining lymph nodes deserve more attention. In addition, memory Th2 cells are thought a key pathogenic cell subset in asthma (25). Thus, we focus on the differentiation of memory Th2 cells in the lung. Memory T cells, including CD4^+^ and CD8^+^ T cells, are generated from the effector pool after the contraction phase rather than directly from the naïve T cell (26, 27). Dyken et al. revealed that local-tissue-checkpoint including IL33, IL25, and TSLP are indispensable for the terminal differentiation of Th2 cells and the concomitant efficient secretion of IL-5 and IL-13 by these Th2 cells (28). Endo et al. found that IL-33 selectively induces IL-5 and ST2 upregulation in memory Th2 cells (29). Significantly enhanced IL-5 and ST2 expression, however, was only observed on memory Th2 cells but not on effector Th2 cells, so it could be speculated that IL-33 plays an instrumental role in the induction of pathogenic memory Th2 cells that execute distinguishing effector program (29). IL-37, a novel described member of the IL-1 family, recently has been shown to curb Th2 immune response by counterbalancing IL-33, representing the potential therapeutic target in asthma (30). Inducible bronchus-associated lymphoid tissue (iBALT), a tertiary lymphoid tissue that develops after inflammation in the airway, contains IL-7 and IL-33-secreting lymphatic endothelial cells (LECs), providing a favorable niche for memory Th2 cells keeping both their survival and pathogenicity (31). IL-2 has previously been known for its role in promoting CD4^+^ T cell proliferation in an autocrine manner (32), however, recently IL-2 has also been shown to be required for memory Th2 cell differentiation and migration to the lung (33). This idea is consistent with an earlier study which found IL-2 is critical for the transformation of effector Th cells into memory Th cells during a rapid contraction phase (34). To explore the mechanism of IL-2 in this process, Bevington et al. found that IL-2 provides the basis for CD4^+^ memory Th cell differentiation *via* establishing an active accessible chromatin landscape (35).

### The differentiation of Th17 cells

The differentiation process of Th17 cells, which is regulated by several cytokines at different stages of differentiation, is more complex than Th1 and Th2 cells. The initial differentiation of Th17 cells is induced by the combination of IL-6 and TGF-β (36). TGF-β is a regulatory cytokine for both Treg and Th17 cells, and IL-6 has a pivotal function in dictating subsequent differentiation of the cells towards the development of Th17 cells *via* inhibiting Treg cell development (36). The IL-17^+^ Foxp3^+^ T cells (Tr17 cells) were identified in experimental autoimmune animal models, indicating that Treg and Th17 cells can interconvert in certain inflammatory conditions (37, 38). It was recently shown that Tr17 cells are induced during allergen-specific immunotherapy (AIT), possibly reflecting a shift of the Th17/Treg balance (39). IL-21 can further propagate Th17 cell differentiation in an autocrine manner when naïve T cells are stimulated by IL-6 and TGF-β (40). Notably, IL-21 can substitute the IL-6 to drive Th17 cell differentiation couple with TGF-β, albeit it is less efficient than IL-6 (41). IL-23 appears to function in late events downstream of Th17 lineage commitment since IL-23R appears not to be induced until after naïve T cells have differentiated into developing Th17 cells in response to IL-6 signaling (42). Akin to IL-23R, IL-1R is induced after Th17 cell differentiation has partially completed *via* IL-6 signaling, but IL-1β reinforces its differentiation at both early and later stages (43). Interestingly, the function of IL-6, IL-21, and IL-23 are all performed through the STAT3 signaling pathway. The timing of expression of specific receptors for three cytokines may determine their respective role.

There is much evidence that the asthmatic lung microenvironment is conductive to Th17 cell differentiation. Some common aeroallergens, including house dust mite (HDM), fungal, and pollen, induce the production of IL-6 from airway epithelial cells (43). TGF-β, a pleiotropic cytokine, has been reported to be constitutively produced by alveolar macrophages (44). In the context of allergic airway inflammation, it is important to note that TGF-β1 drives the Th9 and Th17 cell differentiation while the differentiation of Treg cell is independent of TGF-β1 (45). Exposure to air pollution, especially particulate matter less than 2.5μm that easily reaches small airways and alveoli, *via* interacting aryl hydrocarbon receptor, promotes Th17 cell differentiation to aggravate asthma (46). Respiratory tract colonization with bacteria or viruses occurs in certain patients with severe asthma, which facilitates the differentiation of Th17 cells by regulating the JAK2/STAT3 and Notch1 signaling pathways (47). In the mice model, the airway sensitization route is known to prime mixed Th2-Th17 immune response (48). In this airway sensitization route, a subset of Th17-inducing conventional DCs producing IL-1β has been recently identified in the lung (49).

## The role of Th1 and Th2 cells in the establishment of asthma in early life

The onset of allergic asthma is often in early childhood and how Th1 and Th2 immune responses in this period are influenced may underlie the increased risk of asthma in early childhood. There is a significant transition in the immune system involved in its composition and function during early life. Unlike adults, neonates exhibit Th2 cell skewing, which favors placental growth during pregnancy at the expense of the susceptibility window for asthma (50, 51). This intrinsic skewing is the manifestation of predisposition to the production of Th2-associated cytokines when naïve T cell from a newborn is stimulated *in vitro* (52) However, Th2 cells are scarce in the neonatal lung (53). Thus, shortly after birth, the activation of ILC2s through spontaneous production of IL-33 is at the center of type 2 immunity and the major cellular source of Th2-associated cytokines rather than Th2 cells. Also, spontaneous production of IL-33 during the alveolar phase of lung development induces OX40L and inhibits IL-12 production in postnatal DCs, explaining the Th2 cell skewing in this period (**Figure 2**) (54). Saluzzo et al. found that the activation of the IL-33-ILC2-IL-13 axis from the first day of life shapes the lung immune hemostasis (55). Although type 2 immunity is required for controlling lung development, this skewing decrease the threshold to mount allergen-specific Th2 immune response (56). As newborns are gradually exposed to allergens after the time of weaning, the Th2 immune response will be mounted. In accordance with this notion, Saglani et al. established that the cellular source of IL-13 is mainly from Th2 cells rather than ILC2s for the development of AHR in early life (57). If the newborn lives in an environment full of diverse microbes such as cattle farms, their enhanced Th2 immune response will be suppressed, thus protecting them from the development of allergy and asthma (58). This protective mechanism is achieved by redirecting the Th2 response toward the Th1 response (59). Another protective mechanism is that microbial colonization can decrease hyperresponsiveness towards allergen by inducing Treg cells (60).

In addition to Th2 cell skewing in early life, the impaired primary Th1 immune response also occurs during this period, which offers an opportunity for RV (Rhinovirus) and RSV (Respiratory syncytial virus)-induced lower respiratory illness (61). Since the neonatal ιFN-γ production is impaired and delayed during primary infection compared with adult mice, RV and RSV induce airway epithelial cell necrosis and subsequently lead to the release of IL-33 from the airway epithelium to enhance Th2 immune response (62–64). This finding explains that RV and RSV-induced respiratory infection in early childhood is an independent factor for the later development of persistent asthma (65).

## The role of Th1, Th2, and Th17 cells in stable asthma

Stable asthma is defined as having no recent asthma attack in humans or not in the challenge phase of experimental asthma (66). Compared to asthma exacerbation, stable asthmatic airway inflammation is maintained in relative immune homeostasis. During immune homeostasis, the airway of asthmatic individuals was enriched in Th cell subsets with decreased proinflammatory function. Thus, it is necessary to discuss the role of CD4^+^ T cells during stable asthma and asthma exacerbation separately.

### The role of Th2 cells in stable asthma

Among the various subpopulations of CD4^+^ cells, Th2 cells are most closely associated with the development of asthma. The notion that eosinophilic asthma is a Th2-dominant disease comes from several clinical observations and mouse models. First, several studies have found that increased Th2-associated cytokines, including IL-4, IL-5, and IL-13, were present in the bronchoalveolar lavage (BAL) and blood of allergic asthmatic patients (67–69). Then, this phenomenon also has been demonstrated in an ovalbumin (OVA)-induced asthma mice model, in which hallmark features of asthma are attenuated when OVA-specific Th2 cells are depleted. In contrast, the adoptive transfer of *in-vitro*-generated OVA-specific Th2 cells could induce asthma features (70).

**Figure 3:**
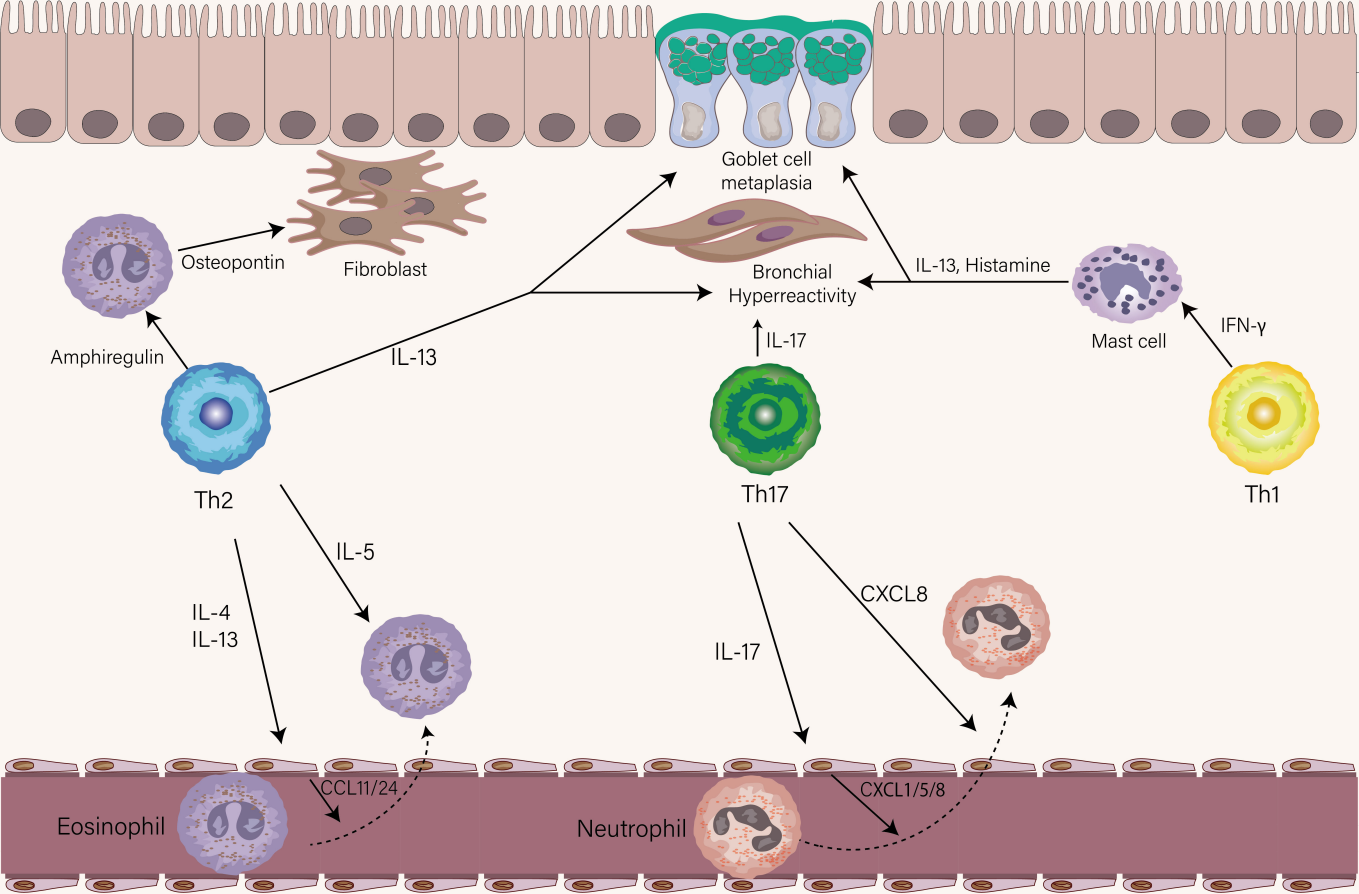
The role of Th1, Th2, and Th17 cells in stable asthma. The eosinophils-recruiting chemokines CCL11 and CCL24 from the endothelial cell are induced by IL-4 and IL-13 from Th2 cells. IL-5 produced by Th2 cells then leads to the activation of eosinophils. Similarly, the neutrophil-attracting chemokines CXCL1, CXCL5, and CXCL8 by endothelial are induced by IL-17 from Th17 cells. Th17 cells could also directly chemoattract neutrophils *via* the release of CXCL8. The production of Amphiregulin and IL-13 is from Th2 cells. Amphiregulin acts on eosinophils to produce osteopontin which triggers airway fibrosis. IL-13 mainly drives goblet cell metaplasia and AHR. IL-17 produced by Th17 cells contributes to the AHR. IFN-γ effects on mast cells increase their ability to release histamine and IL-13, leading to AHR and goblet cell metaplasia.

The cytokines secreted by Th2 cells are mainly IL-4, IL-5, IL-13, and IL-9 which have different roles in the pathogenesis of asthma. IL-4, the polarization factor of Th2 cells, induces antibody class switching in B cells to produce IgE *via* type I receptor (IL-4Rα/γc), which is essential for the initiation of allergic airway inflammation and humoral immune response (71, 72). Recently a study found that IL-13 and IL-4 coordinately, but not IL-4 exclusively, promote induction of high-affinity IgE in humans and mice (18). IL-4 and IL-13 both bind to the type II receptor (IL-4Rα/IL-13Rα1) on the airway epithelium and initiate activation of the STAT6 pathway, whereas the higher affinity of the binding for IL-13 than IL-4 (73). IL-4 is mainly produced by Tfh cells in lymphoid tissue, while IL-13 is mainly produced by Th2 cells in the lung (19). When binding the type II receptor on structural cells of the airway, IL-13 (and to a lesser extent IL-4) causes goblet cell metaplasia, AHR, mucus production, and chemokine secretion including CCL11, CCL24, and CCL26 that help eosinophil recruitment (**Figure 3**) (74, 75). IL-5 is responsible for the maturation, activation, migration, proliferation, and survival of eosinophils that exert their effects in the airway through secreting a series of factors, including Th2-associated cytokines, acute proinflammatory cytokines, and eosinophil-associated proteins (76). The function of the IL-4, IL-5, and IL-13 described above has been elucidated in both humans and mice, however, the function of IL-9 in asthma is still obscure. In murine models of asthma, IL-9 has shown a role in driving AHR, airway remodeling, mast cell survival, and mucous cell metaplasia (77, 78). A recent study found that IL9-producing Th2 cells distinguished from conventional Th2 cells with expression of PPARγ, under the influence of IL-4 and TGF-β, acquire IL-9-producing phenotype (79). Since the transient and heterogenous expression of IL-9 in Th cells, whether Th9 cells, previously recognized as the source of IL-9, represent a distinct T cell in humans is still under debate (80).

It has recently been recognized that the dichotomous pattern of the Th1 and Th2 cells is probably an oversimplification. Advanced technologies have given us deep insight into cell heterogeneity within Th2 cell subsets over time and space. It prompts a reconsideration of whether there were several Th2 cell subsets with a pathogenetic phenotype to respond so proficiently to allergen and to perpetuate recurrent episodes of allergic airway inflammation. Endo et al. proposed “the pathogenic Th-cell disease induction model” to challenge the paradigm that chronic airway inflammation in asthma is induced by overall unbalanced subpopulations of Th1/Th2 cells (25). Those identified pathogenic Th2 cells strongly involved in the pathogenesis of asthma are mainly memory Th2 cells rather than effector Th2 cells (25). In contrast to naive T cells, resting memory T cells are in a primed state that maintains open accessibility to effector molecules genes (79, 81). Memory T cells were divided into two subsets, circulating memory T cells and non-circulating memory T cells, the latter of which is also referred to as tissue-resident memory (TRM) T cells. TRM Th2 cells, a sentinel in the airway that gets reactivated rapidly *in situ* to facilitate an optimally efficient response after re-exposure to allergens, are sufficient to induce some features of asthma (33). As the TRM Th2 cells are enough to induce asthma-like features, raising questions as to whether TRM Th2 cells and circulating memory Th2 cells perform overlapping functions in the lung. Rahimi et al. observed that circulating memory Th2 cells were mainly found around lung parenchyma and TRM Th2 cells preferentially localized near the airway. The different tissue localization explained that TRM Th2 cells are responsible for peribronchial inflammation, whereas circulating memory Th2 cells mainly promote perivascular inflammation (82).

Recent studies identified several memory Th2 cell subpopulations with distinct phenotypes or gene signatures. In a murine model, IL-5 high-producing Th2 cells were demonstrated to be a key pathogenic population in the pathogenesis of eosinophilic airway inflammation (25). Also, this Th2 cell subset was induced in multiple rounds of *in vitro* differentiation, suggesting a highly differentiated phenotype (83). Based on the expression of CD161, CRTH2, and hPGDS, another dual-IL5/IL13 high-producing-pathogenic Th2 cell named peTh2 cell was identified in the blood of patients with allergic eosinophilic inflammatory diseases (11). A clinical study performed on allergic patients showed that the Th2A cell subset in peripheral blood was characterized by higher expression of *IL17-RB* and *IL1R1* and higher production of IL-5 and IL-9 relative to conventional Th2 cells (11). A recent study using single-cell transcriptomics identified a subset of pathogenetic Th2 cells in the airway of asthmatic individuals characterized by high expression of *IL4, IL5, IL13, IL17RB, HGPDS*, and *PPARG* (**Figure 1**) (84). Unlike eosinophilic inflammation, airway remodeling, a result of long-lasting allergen exposure, correlates with asthma severity and resistance to corticosteroids (85). Morimoto et al. found that amphiregulin-producing memory Th2 cells are central to the induction of airway fibrotic responses *via* the osteopontin produced by eosinophils (**Figure 3**), extending previous observations of such pathogenetic Th2 cells by their group (86). In recent work, the novel TRM Th2 cells identified as CD69^hi^ and CD103^lo^ population have a unique phenotype of exhibiting enhanced effector cytokines production such as IL-5, IL-13, and high levels of fibrosis-related genes including *Areg* in mouse models of allergic inflammation (87).

Whether the above pathogenetic Th2 cells perform overlapping functions or whether each cell subpopulation can play a different role in the pathogenesis of asthma is still an open question. Previous studies showed several pathogenetic Th2 cell subsets, as assessed by sustained high expression of one or two cytokines. The complexity of Th cell cytokine expression patterns far exceeds our expectations by using mass cytometry or cytometry by time of flight (CyTOF), so the cytokine footprints of these identified pathogenetic Th2 cells warrant further investigation. Moreover, the cellular mechanisms that control the generation of pathogenetic Th2 cells are needed to elucidate.

### The role of Th1 cells in stable asthma

For a long time, Th1 cells were thought to play a protective role in asthma. An early study has shown that administration of IL-12, the key Th1-polarized cytokine, inhibits AHR and eosinophilia in murine models of eosinophilic asthma (1). The primary mechanism of AIT for allergic asthma is thought the shift from Th2 to Th1 response, accompanied by an increase in IFN-γ levels and a decrease in IL4 levels (88). Secretoglobin1A1 (SCGB1A1), a negative mediator for Th2 immune response, has been reported to be induced in the upper and lower airway in patients treated with AIT (89, 90). IFN-γ may partly mediate this process by inducing SCGB1A1 production from the airway epithelium (91). Th1/Th2 antagonism is also reflected in epithelial antagonistic gene regulation through IFN-γ and IL-4. Zissler et al. found that IFN-γ could attenuate IL-4-induced genes that promote allergic airway inflammation and airway remodeling (92). Subsequent work in the same group found that upper-airway IL-24, an IL-4-induced epithelial type 2 cytokine, could be used as a proxy for lower-airway IL-24 to diagnose allergic asthma (93). Recently, Cautivo et al. proposed a topographic cross-regulation model for Th1 and Th2 cells in the lung, wherein IFN-γ production by Th1 cells directly restricts IL-5-producing Th2 cells dispersion and lung parenchymal accumulation (94). The evidence mentioned above supports the original Th1/Th2 paradigm that described a simple framework for our understanding of protective and pathogenetic response in the context of CD4^+^ T cells in response to the allergen.

Perplexingly, there is mounting evidence on the pathogenetic role of Th1 cells in patients with severe asthma and mouse model. In a double-blind, randomized, parallel-group clinical study, the administration of recombinant human IL-12 to allergic asthmatic patients had not any significant effects on AHR (95). Using an adoptive transfer system, Hansen et al. found that OVA-specific Th1 cells unexpectedly failed to counterbalance Th2-dominated airway inflammation and AHR but cause severe airway inflammation (96). The serum levels of IFN-γ have been reported to be positively correlated with AHR in allergic asthmatics (97). In another similar report, elevated numbers of virus-specific Th1 cells correlated with worse lung function in asthmatic subjects (98). Analysis of BAL among severe asthmatics has found dominant Th1-associated cytokine expression profiles and neutrophilic inflammation (3, 99, 100). Studies of transcriptomic with an unbiased clustering approach in asthmatic patients unveiled a distinct phenotype with increased expression of Th1 signature genes (101, 102). Several clinical studies have reported that severe asthmatic patients with Th1-dominated inflammation had a positive result for microbial organisms or viruses, which indicates respiratory pathogens are potent triggers for driving Th1 immune response in these patients (3, 103, 104). Of note, these patients had no signs of current or recent active infection. We thus raise the possibility that colonization of pathogens after infection in the airway produces a favorable milieu for maintenance and chronic reactivation of the virus and bacteria-specific Th1 cells that are integral to severe asthma pathogenesis. Although a critical question regarding a definitive role for the contribution of Th1 cells in asthma remains unclear, several important underlying mechanisms required for Th1-mediated pathogenic response have been elucidated. Deeper insight into the IFN-γ-mediated AHR *in vivo* was gained by Raundhal et al. using a Th1-dominated murine model that could reflect severe human asthma (103). It is possible that the causal relationship between the expression of high levels of IFN-γ and lower secretory protease leukocyte inhibitor (SLPI) expression, contributes to AHR. SLPI was identified as a potent inhibitor of tryptase that activates protease-activated receptor 2 (PAR-2) to cause AHR (105). One of the hallmark features of severe asthma is resistance to corticosteroids and IFN-γ is associated with a poor response to corticosteroids (103). In the isolated airway smooth muscle cell experimental system, two studies have demonstrated that a combination of IFN-γ and TNF-α promotes corticosteroid resistance through the upregulation of glucocorticoid receptor beta isoform (GRβ) and IRF-1, respectively (106, 107). Gauthier et al. found that upon administration of corticosteroid, IFN-γ induced STAT1 binding to critical regulatory elements within the *CXCL10* promoter was not inhibited. However, the enrichment of corticosteroid receptor for the same gene site has been observed, which resulted in slightly increased transcription levels of *CXCL10* (108). In contrast to IFN-γ, corticosteroids suppressed LPS-induced *CXCL10* gene expression, thus establishing the IFN-γ-CXCL10 axis as a critical player in corticosteroid-refractory asthma. IFN-γ-mast cell axis was identified as a pathway involved in crosstalk between Th1 and Th2 immune response in a mice model of chronic asthma. In this axis, IFN-γ is required for optimal IgE-dependent release of mediators that include IL-13 and histamine (**Figure 3**) (109).

### The role of Th17 cells in stable asthma

Th17 cells and IL-17A are upregulated in the lungs of patients with asthma, especially individuals with severe asthma, suggesting Th17 cells are involved in the pathogenesis of moderate to severe forms of asthma (110, 111). Both in human and mouse experimental asthma models, IL-17 orchestrates neutrophil recruitment to the lung either directly through CXCL8 production or indirectly through promoting the release of CXCL8, CXCL1, and CXCL5 by structural cells (**Figure 3**) (112, 113). The contribution of IL-17 to airway remodeling during asthma includes inducing airway smooth muscle and fibroblast proliferation (114). IL-17 can break the Treg/Th17 balance, which alleviates the anti-inflammatory role of Treg cells (115). Treatment with dexamethasone *in vitro* inhibits cytokine production by Th2 cells but has no effect on IL-17 production by Th17 cells, indicating that Th17 cells may induce steroid-insensitive phenotype (116). The role of Th17 cells in steroid-insensitive asthma has been further corroborated in the mice model, in which the adoptive transfer of allergen-specific Th17 cells to mice or overexpressing RoRγt in transgenic mice develop a steroid-refractory neutrophilic asthma model (116, 117). In human studies, IL-17 has been shown to be involved in steroid-insensitive asthma through upregulation of the expression of GRβ in peripheral mononuclear cells (118). The role of Th17 cells in mediating AHR has long been controversial. IL-17 has been reported to cause smooth muscle cell contraction directly and independently contribute to AHR in a mice model of steroid-insensitive airway inflammation (**Figure 3**) (119, 120). In asthmatic patients, the levels of IL-17 are positively correlated with AHR (121). In contrast, this notion failed to be corroborated in an experimental mice model of severe asthma with Th1 and Th17 properties, in which IFN-γ, but not IL-17, is responsible for the development of AHR (103). A possible hypothesis is that the functionality of Th17 cells depends on the context of the combination of different subsets of Th cells. In addition to IL-17, IL-22 has initially been thought a signature cytokine for Th17 cells, but subsequent studies identified a previously unknown subset of human Th22 cells that produce high levels of IL-22 without IL-17 production (122).

### Relationship between Th2 and Th17 cells in asthma

The reciprocal relationship between Th2 and Th17 cells has been favored on the basis of reduction in IL-17 expression mediated by IL-4 *in vitro* experiment (8). This relationship was also supported by experimental allergic asthma, in which blockade of Th2-associated cytokines enhances Th17 immune response whereas blockade of IL-17 augments Th2 immune response (2). However, increasing evidence suggests that Th2 and Th17 immune responses are coexisting and more tightly interconnected than anticipated. Treatment with IL-6 monoclonal antibody or RoRγt inhibitor attenuates both Th2 and Th17 immune response in mice, resulting in reduced neutrophilic and eosinophilic inflammation in the airway (123, 124). Using the HDM-inducing model of asthma, Izumi et al. found that the differentiation of Th2 and Th17 cells *in vivo* is mounted by the same lung conventional CD11b^+^ DCs (cDC2) at different phases of maturation (49).

Although historically thought to be derived from conventional Th17 cells, it is now understood that IL-17 is mainly produced by IL-17-producing Th2 cells that co-express ROR-γt and GATA-3 with the capacity to simultaneously produce both IL-4 and IL-17 during the chronic stage of asthma (**Figure 1**) (125). It has been shown that *in-vivo*-generated OVA-specific IL-17-producing Th2 cells could transfer the inflammatory effect so that both eosinophils and neutrophils were recruited into the airway (125). A higher frequency of IL-17-producing Th2 cells was found in the blood and BAL of subjects with severe and corticoid-resistant asthma (125, 126). IL-17-producing Th2 cells are characterized by higher levels of IL-4 production and increased expression of MEK that inhibits dexamethasone-induced cell death (126). This can explain why IL-17-producing Th2 cells induce more severe asthma when compared to conventional Th2 and Th17 cells (125, 126). The generation of IL-17-producing Th2 cells is still unclear because conventional Th2 cells had the potential to produce IL-17 in certain cytokine milieu (125). Meantime, Th17 cells were also predisposed to acquire a Th2-associated cytokine expression profile when the inflammatory conditions changed (127). In the absence of IL-4 and IFN-γ, naïve T cells stimulated with IL-21, IL-1β, and IL-6 can differentiate into IL-17-producing Th2 cells (128). The unstable and plasticity of Th17 cells, which lead to upregulation of Th2-associated cytokine, was examined in detail using the IL-17A reporter system in the HDM-driven model of asthma (127). An alternative possibility is that the complex *in vivo* priming conditions may drive naïve T cells to skip Th2 or Th17 cell differentiation and directly induce the differentiation of pathogenetic Th cell subsets with both Th2 and Th17 functionalities. As Th17 cells show more functional plasticity and instability than Th2 cells, we propose a hypothesis that the influx of the initial polarization of Th17 cells into the lung is not stably committed at the challenge phase of allergic asthma and subsequently converts into more pathogenic IL-17-producing Th2 cells that sustain persistent mixed eosinophilic and neutrophilic inflammation during the chronic stage of asthma. Moreover, IL-17-producing Th2 cells in the context of Th2-associated milieu convert into IL-17-negative Th2 cells that positively correlate with neutrophilic but not with eosinophilic inflammation in the airway (127).

### Relationship between Th1 and Th17 cells in asthma

IL-17 was initially believed to be produced by IFN-γ^+^ τh1 cells, and IL-23 was responsible for both induction of IL-17A and IFN-γ expression, indicating a developmental relationship between Th1 cells and Th17 cells (129). The early report has identified the relationship between Th1 and Th17 cell-mediated immunity in the autoinflammatory disease model wherein IL-23 and IL-12 promote Th17 cells converted to pathogenetic IFN-γ-secreting Th17 cells (130). Recently, the mingling of Th1 and Th17-associated cytokine expression patterns also manifest in asthma, as demonstrated by the presence of IFN-γ^+^ IL-17A^+^ Th cells in the lower airways of children with severe treatment-refractory asthma (**Figure 1**) (3). IL-17 and IFN-γ have been reported to play a role in mediating neutrophilic inflammation and AHR, respectively, which suggests that IFN-γ^+^ IL-17A^+^ T cells exhibit a more significant pathogenetic effect than conventional Th1 or Th17 cells (103). IL-23 as a member of the IL-12 cytokine family is beneficial to the induction of IFN-γ^+^ IL-17A^+^ T cells. Higher serum IL-23 levels were found in asthmatic subjects versus healthy subjects and correlated with greater impairment in lung function (131). In the absence of IL-23, Th17 cells differentiated by IL-6 plus TGF-β produce both IL-17 and IL-10, the latter of which confer Th17 cells a protective role rather than a pathogenic role in the model of autoimmune diseases model (132). It is surprising that despite being reported to inhibit both Th1 and Th17 immune responses, Risankizumab, the monoclonal anti-IL-23 antibody, had no beneficial effect on severe asthma (133). Risankizumab attenuates expression of the Th1 and Th17 transcriptional factor in sputum from asthmatic patients and was also shown to have marked clinical efficacy in Th1 and Th17-associated diseases like psoriasis and Crohn’s disease (133–135). One possible explanation is that inhibition of IL-23 enhances Th2-dependent eosinophilic inflammation due to the postulated antergic relationship between Th2 cells and Th1/17 cells. The other possible explanation is that this Risankizumab clinical strategy did not specifically enroll patients with severe asthma on the basis of up-regulated IL-23 or Th1/Th17 signature. Therefore, the notion that IL-23 as a new therapeutic strategy to diminish both Th1 and Th2 pathways obtained from the model of autoimmune diseases also holds true in the case of severe asthma warrants further investigation.

## The role of Th1, Th2, and Th17 cells in asthma exacerbation

Respiratory viral infections (mostly caused by RV and RSV) are the most common cause of asthma exacerbation. Synergistic interaction between respiratory viral infection and allergen exposure has long been recognized to increase the risk of asthma exacerbation (136).

**Figure 4:**
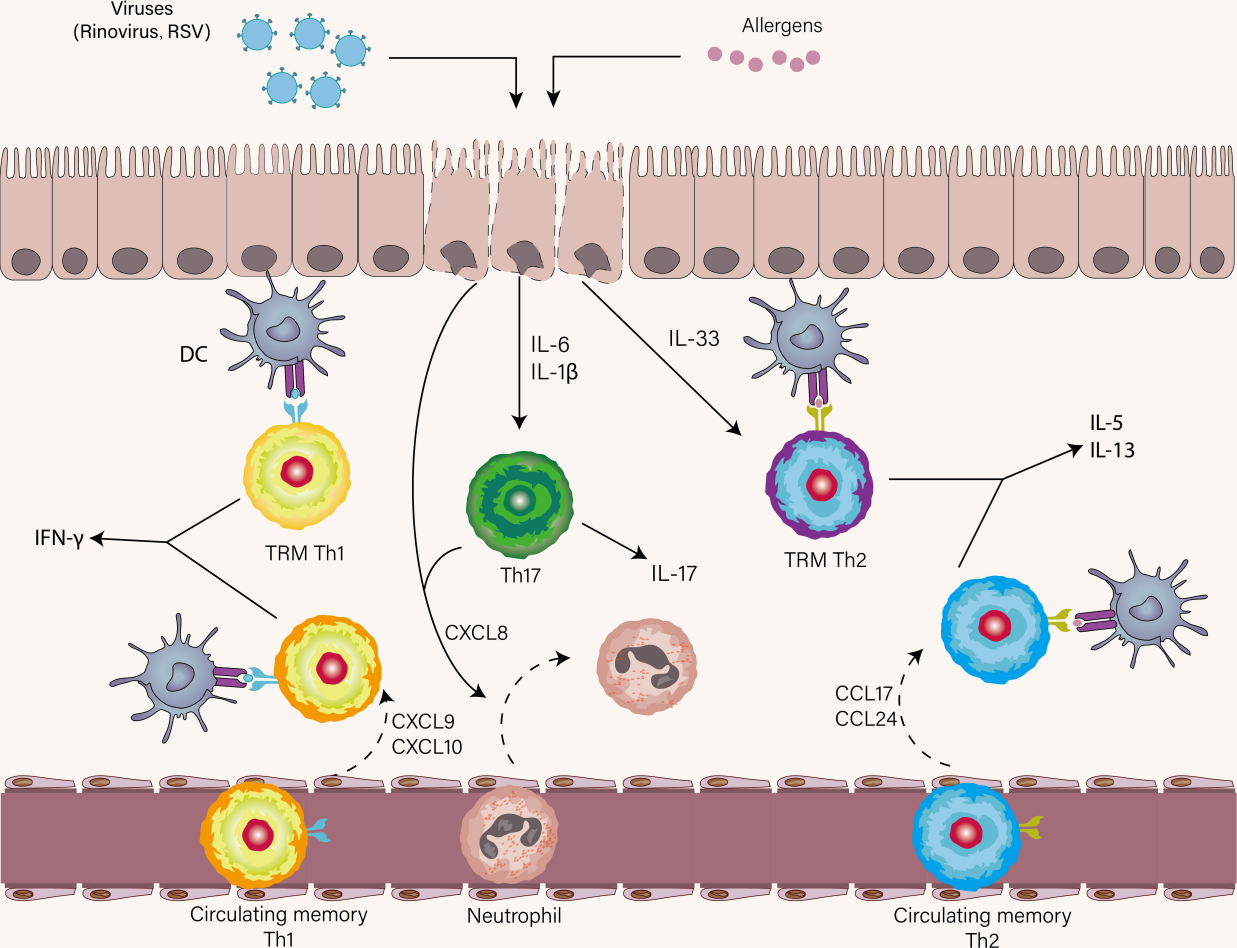
The role of Th1, Th2, and Th17 cells in asthma exacerbation. Once encounter respiratory viruses and allergens, airway epithelial cells produce a wide range of cytokines as the first immune responders, most notably IL-33, IL-1β, IL-6, and CXCL8. Respiratory viral infections mostly elicit a Th1 immune response and less commonly trigger a Th17 immune response. TRM Th2 cells are first reactivated in the presence of IL-33 and cognate antigen. Subsequently, circulating memory Th2 cells are recruited to the lung. A similar scenario also occurs in virus-specific Th1 immune response. IL-1β and IL-6 promote Th17 cell differentiation and CXCL8 derived from Th17 cells and epithelial cells induce neutrophil recruitment to the lung.

Airway epithelial cells are the first immune responder to viruses and allergens, both of which result in the release of IL-33, IL-6, IL-1β, and CXCL8 by epithelial cells (**Figure 4**) (137). As already mentioned, IL-33 is pivotal to the terminal differentiation of Th2 cells and reactivating memory Th2 cells independently of TCR stimulation. Asthma patients who are prior exposed to allergens have increased memory allergen-specific Th2 cells with enhanced ST2 expression. This can explain that amplified Th2 immune response in response to IL-33 was present in asthmatic patients with experimental RV infection compared with healthy volunteers (138). In a similar study, the rapid expansion of allergen-specific Th2 cells and increased production of type2 cytokines were found in the nasal lining of individuals with RV-induced asthma exacerbation, indicating bystander activation and proliferation of these Th2 cells might occur in the absence of cognate antigen (98). However, it is important to note that IL-33 failed to induce memory Th2 cell expansion and Th2-associated cytokine expression alone in the HDM model of allergic airway disease, suggesting memory Th2 immune response was an antigen-dependent process (82). These findings establish a link between viral infection and amplified Th2 immune response, and IL-33 acts as a bridging mediator of this link. Moreover, a seminal study has reported that RV-induced neutrophil extracellular traps (NETs) boost Th2 immune response during Th2 cell priming, thus promoting asthma exacerbation (139). Taken together, for Th2 immune response in asthma exacerbation, TRM Th2 cells are the earliest Th2 subsets to respond proficiently to allergens by robustly initiating expansion and effector program execution. Following the reactivation of TRM Th2 cells, circulating memory Th2 cells are recruited into the lung by CCL17 and CCL24, and then Teff2 cells are primed on DCs in lymphoid tissue (**Figure 4**) (139).

Respiratory virus infection mainly induces Th1 cells, especially TRM Th1 cells which may be crucial in secondary encounters with viruses. Virus-specific TRM Th1 cells are reactivated rapidly in the airways following RSV experimental infection of individuals (140). Their immediate production of IFN-γ facilitates the recruitment of circulating memory Th1 cells (**Figure 4**) (141). Several earlier works demonstrated impaired Th1 immune response in asthmatic patients (142–144). This notion comes from (1) *in vitro* cellular data showing that both peripheral blood and BAL of Th1 cells from asthmatic subjects challenged by RV induce lower levels of IFN-γ versus normal subjects (142) (2). a link between lower expression of IFN-associated genes and shorter time to exacerbation (145). These studies suggest that preexisting Th2 inflammation increases the threshold to mount Th1 immune response. Reciprocally, the attenuation of Th1 immune response in asthma was not supported by other work, in which some have shown induction of type1 or type2 IFN at similar levels in asthmatic patients and controls (98, 146, 147), whereas others even have shown excessive IFN-associated response in asthmatics compared with controls (148, 149). According to the kinetics of IFN response *in vivo*, we speculate that impaired Th1 immune response at baseline favors viral replication during early acute infection, which paves the way for a subsequent exaggerated Th1 immune response promoting exacerbation. Collectively, Th1 cells can sometimes protect and sometimes favor asthma exacerbation depending on the time of infection. The conventional view is a mutually antagonistic relationship between Th1 and Th2 cells. Randolph et al. reported that the accumulation of Th2 cells in the lung was aided by Th1 cells in a mice model of acute eosinophilic airway inflammation, which challenged this view (150). Subsequently, using an experimental infection model in both humans and mice, virus-specific Th1 cells have shown to potentiate the recruitment of resting Th2 cells, suggestive of the cooperation between Th1 and Th2 cells in asthma exacerbation (98, 151).

Under the influence of IL-6 and IL-1β, Th17 cells are induced and subsequently release CXCL8 with epithelial cells to recruit neutrophils (**Figure 4**). The neutrophil recruitment occurs in the early stage of viral infection. Most virus-primed CD4^+^ T cells are Th1 subsets and to a lesser degree Th17 subsets (152). In addition, virus-induced type I IFN inhibits the Th17 cell differentiation, thus the role of Th17 cells during virus-induced asthma attacks seems to be negligible (153). The inhibition of IL-17A production in Th17 cells by an RV infection in the setting of asthmatic children and experimental mice asthma lends further credence to this notion (154). In contrast, during asthma exacerbation, another work has found increased levels of IL-17A from nasal lining fluid and increased frequency of Th17 cells in peripheral blood of asthmatic patients (66, 98). One of the characteristics of those who had virus-driven asthma exacerbation was high sputum neutrophils that have shown to skew the polarization of naïve T cells to Th17 cells *via* neutrophil antimicrobial peptide cathelicidin (155, 156). A recent prospective study identified a causal relationship between circulating IL-6 and asthma exacerbations, which could be plausibly explained by IL-6-driven differentiation of naïve T cells into Th17 cells that trigger asthma exacerbation (157). The genetic polymorphism in the *IL-4Rα*, which is associated with asthma exacerbation (158), potentiates the Th17 immune response in an IL-6-independent manner (159). These findings lend further credence to the role of Th17 cells in asthma exacerbation. A caveat in the studies mentioned above is that the inflammatory profile of exacerbation did not all fit virus-induced criteria.

Collectively, we mainly describe the asthma exacerbation caused by cooperation between viruses and allergen exposure in this part, especially RV. Respiratory viral infection potentiates ongoing Th2 response meantime preexisting Th2 inflammation attenuates antiviral response but elicits a dysregulated Th1 immune response.

## Conclusion

In this review, we highlighted the inflammatory timeline of asthma pathogenesis from inceptive, chronic to acute process. In this timeline, we focus on the differentiation and function of Th1, Th2, and Th17 cells and their mutual crosstalk. It should be noted that although the main source of effector cytokine is innate cells like ILC2 in some contexts, the precise timing of differentiation, tissue migration, and activation of CD4^+^ T cells may unveil a clearer visualization of the cellular and molecular mechanism that control the pathogenesis of asthma. As heterogeneity and plasticity of Th cell subsets mentioned in the review only represent a part of immune cells involved in asthma pathogenesis, whether these so-called pathogenetic Th subsets and other immune cells display functional redundancy is still an open question. In addition, how complex cues *in vivo* regulate the generation of pathogenetic Th subsets or non-pathogenetic Th subsets warrants further investigation.

## Author contributions

WL drafted the manuscript and designed the figures. JD, JH, and WX revised and provided expert opinions on the manuscript. All authors contributed to the article and approved the submitted version.

## Funding

This work was supported by grants from the National Natural Science Foundation of China (82174170, 8217141327), Shanghai Science and Technology Commission (grant No.18401901800, 21S21902500), and Expert Workstation for Jingcheng Dong in Yunnan Province (202110101).

## Acknowledgments

The authors greatly thank Xie Cong and Wenjing Chen from Fudan University for guidance on the images.

## Conflict of interest

The authors declare that the research was conducted in the absence of any commercial or financial relationships that could be construed as a potential conflict of interest.

## Publisher’s note

All claims expressed in this article are solely those of the authors and do not necessarily represent those of their affiliated organizations, or those of the publisher, the editors and the reviewers. Any product that may be evaluated in this article, or claim that may be made by its manufacturer, is not guaranteed or endorsed by the publisher.
